# Algal Toxic Compounds and Their Aeroterrestrial, Airborne and other Extremophilic Producers with Attention to Soil and Plant Contamination: A Review

**DOI:** 10.3390/toxins13050322

**Published:** 2021-04-29

**Authors:** Georg Gӓrtner, Maya Stoyneva-Gӓrtner, Blagoy Uzunov

**Affiliations:** 1 Institut für Botanik der Universität Innsbruck, Sternwartestrasse 15, 6020 Innsbruck, Austria; georg.gaertner@uibk.ac.at; 2 Department of Botany, Faculty of Biology, Sofia University “St. Kliment Ohridski”, 8 blvd. Dragan Tsankov, 1164 Sofia, Bulgaria; mstoyneva@uni-sofia.bg

**Keywords:** algae, caves, cyanobacteria, cyanotoxins, cyanopeptides, deserts, human health risk, hypersaline habitats, phycotoxins, thermal springs

## Abstract

The review summarizes the available knowledge on toxins and their producers from rather disparate algal assemblages of aeroterrestrial, airborne and other versatile extreme environments (hot springs, deserts, ice, snow, caves, etc.) and on phycotoxins as contaminants of emergent concern in soil and plants. There is a growing body of evidence that algal toxins and their producers occur in all general types of extreme habitats, and cyanobacteria/cyanoprokaryotes dominate in most of them. Altogether, 55 toxigenic algal genera (47 cyanoprokaryotes) were enlisted, and our analysis showed that besides the “standard” toxins, routinely known from different waterbodies (microcystins, nodularins, anatoxins, saxitoxins, cylindrospermopsins, BMAA, etc.), they can produce some specific toxic compounds. Whether the toxic biomolecules are related with the harsh conditions on which algae have to thrive and what is their functional role may be answered by future studies. Therefore, we outline the gaps in knowledge and provide ideas for further research, considering, from one side, the health risk from phycotoxins on the background of the global warming and eutrophication and, from the other side, the current surge of interest which phycotoxins provoke due to their potential as novel compounds in medicine, pharmacy, cosmetics, bioremediation, agriculture and all aspects of biotechnological implications in human life.

## 1. Introduction

The term “algae” has no taxonomic standing but is routinely used to unify the thallic phototrophs with size range from 0.2 µm to more than 75 m, which channelize solar energy through photosynthesis and produce oxygen [1]. Algae are a heterogenous group of polyphyletic origin, which due to their prokaryotic and eukaryotic cell organization belong to two domains of the Tree of life, Eubacteria and Eukarya [1,2,3]. Much of them inhabit the classical ambient aquatic environments (e.g., lakes, ponds, bogs, dams, rivers, seas, oceans), where they live on the bottom (benthos) and in the water column (plankton) [1,4]. However, algae are hardy and occur also in many other diverse places on our Planet. They include soils and subaerial solid substrates (e.g., tree barks, roots and leaves, rocks, walls of city buildings, industrial constructions and historical monuments), and some other, like caves, snow, ice and thermal springs, which are beyond the normal limits for growth and have been long considered inhospitable for living organisms [4,5,6]. These inimical environments on the edge of temperature, pH, salinity, water supply, light, high metal content or radiation, are hostile and even lethal for most life forms and are classified as extremophilic [5,6,7,8,9,10]. To this group belong also some aquatic habitats, such as acidic lakes, alkaline lakes, humic lakes, hypersaline waterbodies, etc., which also offer harsh conditions to survive [11]. After the term extremophile was coined by Macelroy in 1974 [12] for organisms that are able to reproduce at or near to the extreme ranges of individual environmental variables, it became obvious that some of them have to withstand even more harsh life under multiple stress factors [13]. These organisms were named polyextremophiles [10,13,14].

However, all these terms and classifications are not exclusive. For example, soil and subaerial algae are often united as a common ecological group named aeroterrestrial algae [15], but some authors consider them extremophiles because of their life on the boundary between solid substrate and air [16,17]. To cite more examples, there are opinions that waterbodies at the extremes of nutrient content could be referred among extreme habitats [7] but generally other stresses and extremes that can be more easily measured and evaluated, are considered extremophilic [13]. Additionally, there is a group of so-called airborne algae (aeroplankton [18]), which includes diverse freshwater, marine, soil, subaerial and other algae which can be lifted in the air and driven by the wind [19,20]. Clouds have traditionally been not accepted as a permanent environment since they have been thought to be too hostile and too short lived. By contrast with this prior view, nowadays, data are accumulated that they contain organisms (in both active and dormant stages) from different groups (bacteria, algae, fungi, protists) and can also be regarded as extreme habitats [13].

Because of the limited scope of the review, we shall use the terms related with extremophiles in their broadest sense, without commenting in detail the differences between organisms, which are “true lovers” (-philes) of such conditions and organisms which can only tolerate them. Due to the same reason, we do not discuss the unique extreme habitats, from which algae have not been reported (e.g., the isolated Blood Falls of Antarctic [7], or in which potentially toxic algae were not found (e.g., the columns in Ikka Fjord, Greenland [13,21,22]). Readers are also kindly invited to check the references from the cited publications, which we tried to reduce to a reasonable minimum.

Currently, the aeroterrestrial algae, extremophiles and polyextremophiles from all unusual and inhospitable for humankind places attract the attention of scientists in a raising way. Although all strategies to survive in harsh environments are not fully clarified, it is widely accepted that their inhabitants have developed a variety of coping mechanisms, many biomolecules and peculiar biochemical pathways, the interest to which is constantly growing not only in respect to ecology and astrobiology but also for innovative needs of biotechnology, medicine, pharmacy and cosmetics, and all related economical activities including tourism [5,7,8,9,14,23,24].

Algae have played crucial role in development of the global ecosystem, starting with changing the ancient Earth atmosphere, due to their combined ability for oxygen production and for fixation of atmospheric nitrogen (N_2_) in energy expensive reduction to ammonium (NH_4_^+^) [25]. These two processes were accomplished in a unique way by the earliest prokaryotic blue-green algae (currently known as Cyanoprokaryota/Cyanobacteria), “the largest, most diversified, and ecologically most successful prokaryotic organisms on Earth” [25] (p. 141), which appeared in Early Proterozoic [26]. Cyanoprokaryotes were not only the phototrophic pioneers on the planet, but also today they are the first algal inhabitants of new environments, like newly erupted islands, post-volcano environments and open surfaces, where they have no real competitors at least in the moment of their appearance [4,5]. Furthermore, cyanoprokaryotes were significant players in the evolution scene as plastid progenitors important in the Earth land conquest. At present, it is believed that evolution of the land plants from a green algal ancestor was the key event in the history of the terrestrial environments [27]. This happened after the eukaryotic host cell engulfed a photosynthetic cyanoprokaryote and used it as a plastid in the process of primary endosymbiosis [1,28]. The three major resulting evolutionary lineages are the green algae (Chlorophyta and Streptophyta), red algae (Rhodophyta) and glaucocystophytes (Glaucocystophyta) [1,28]. Multiple endosymbioses led to the appearance of the next evolutionary lineages of divisions (phyla) Euglenophyta, Chlorarachniophyta, Cryptophyta, Pyrrhophyta, Haptophyta and Ochrophyta [1,28]. In this way, for more than two billion years, algae (with lichens on latter stages), before appearance of land plants, were the sole contributors to terrestrial organic carbon pools [29,30].

At the same time, it has been reported extensively that cyanoprokaryotes are among the most dangerous organisms of the Earth, which produce numerous toxic compounds that can detrimentally affect human and ecosystem health with most accounts related to bloom-forming species in freshwater and shallow coastal waters [31,32]. Toxigenic algae have been identified also in aquatic habitats in some of their eukaryotic descendants from the divisions Pyrrhophyta, Haptophyta, Ochrophyta (classes Bacillariophyceae and Raphidophyceae), Euglenophyta, Rhodophyta and Chlorophyta [31,33]. General consensus exists that the main exposure routes are through contaminated water, consumption of poisoned food, respiration of infested aerosols, or direct skin contacts during swimming or other recreational activities, and accidental exposure through dialysis water has been evidenced [31,32,34]. Routinely the toxic effects are associated with mass developments of algae (blooms), but some species can cause harm at low concentrations. For example, *Alexandrium tamarensis* can affect shellfish in amounts of 10^3^ cells L^−1^ [35].

Algal toxins (phycotoxins) have been reportedly implicated in health problems and are well-known for being of great concern for humans and ecosystems, which can be of military interest also [36,37,38]. However, nowadays, these natural compounds are increasingly seen positively as potential new biotechnological tools like biocides in agriculture and as potent compounds in medicine and pharmacy with strong antitumor, antibacterial, antiviral, antihelminth and other activities [16,31,39,40,41,42,43,44,45]. This dualistic nature of phycotoxins provokes surge of interest to their structures, origin, real role(s) in producing cells, effects on other organisms, as well as to driving forces of their production and future prospects related with increased anthropogenic impact and global climate changes.

Toxigenicity is not a ubiquitous feature at generic and species level: both toxic and non-toxic strains have been isolated from natural populations of the same species and coexist in different environments (mainly waterbodies) around the world [32,46]. Considering the great phototrophic role of algae, and of cyanoprokaryotes in particular, as pioneers of almost all land habitats, a logical question is whether there is a strong difference in the spread and role of toxic aquatic algae, on one hand, and their aeroterrestrial and extremophilic counterparts, on the other. It was supposed also that the study of toxin production in extreme environments may help in understanding their functional role [47], which is yet not fully understood. Interest in soil and plant contamination by algal toxins has grown significantly after numerous observations have shown that phycotoxins may be accumulated by plants and transferred throughout food webs to other trophic levels [48]. However, due to meager literature concerning toxins in aeroterrestrial and extreme habitats [49,50], finding answers at the moment is very difficult.

In order to gain a complete picture on the current knowledge on versatile toxic compounds and their producers in these environments, we analyzed the available literature and provide an outlook on phycotoxins and toxigenic algae in aeroterrestrial and extreme habitats related with their peculiar ecology and potential route exposures. We believe that this review will help to clarify the current state-of-art on the topic and to identify gaps in knowledge that should be addressed in future research. The understanding of toxins spread can help better to understand the role of these compounds, to predict some perhaps yet hidden effects, to maintain better ecosystem management and prevent health risks, but also will draw attention to the possible application of the algae from these groups in different spheres of human life like medicine, cosmetics, biotechnology, the surge of interest to which is currently seen.

## 2. Main Types of Toxic Algal Compounds

Different approaches were used in classification of toxic algal compounds and beyond those, which are chemically and structurally well-determined and ultimately named toxins, many extracts or exudates have displayed allelopathic or other detrimental effects in laboratory tests and experiments [31,32,51]. In this review we consider only chemically defined toxic compounds produced by algae, the toxicological profiles of which raised concerns on human, animal and ecosystem health. In this way, we discuss not only the routinely known “standard” phycotoxins, such as microcystins, saxitoxins, etc., but also some other metabolites with pronounced cytotoxic and other inhibiting properties. However, we skip data on the hazardous effects of crude or other extracts without chemically identified toxins (e.g., [52,53]) and eliminate a large number of data related with antiviral, antibacterial and antifungal activity, as well as on allelopathic effects on other algae and plants. We also do not discuss pathogens for which no indication of released toxins is available (e.g., the green parasitic algae *Prototheca*, which cause prototecosis in humans, dogs and cattle [54,55,56]). Moreover, we eliminated data on some biologically active compounds, produced by algae and known for their enormous biotechnological and medical importance, which could be toxic to other organisms. For example, the polyunsaturated fatty eicosapentanoic acid (EPA) released by benthic diatoms during grazing can be acutely toxic to the freshwater anostracan grazer *Thamnocephalus platyurus* [57].

The toxins produced by Cyanoprokaryota (cyanotoxins) are commonly combined in four groups according to the main target of their action: (1) neurotoxins (anatoxins—ATXs/incl. anatoxin-a—ATX, homoanatoxin-a—HTX, etc./, guanitoxin—GTX, saxitoxins—SXTs, β-Methylamino-l-alanine—BMAA and its two main isomers 2,4-diaminobutyric acid (2,4-DAB) and N-(2-aminoethyl)glycine—AEG); (2) cytotoxins (cylindrospermopsins—CYNs); (3) hepatotoxins (microcystins—MCs, nodularins—NODs); (4) dermatotoxins (lyngbyatoxins—LAs, aplysiatoxins—ATs, debromaplysiatoxins—DATs, anatillatoxin) [32,58]. Chemically they are classified as peptides (oligopeptides, cyclic peptides, or non-ribosomal peptides—NRPs) (MCs and NODs), alkaloids (ATXs, SXTs, CYNs, LAs, DATs, anatillatoxin), phosphorylated cyclic N-hydroxyguanine (GTX, formerly known as ATX-a(S) [58]), diaminoacids (BMAA, DAB, AEG), and lipopolysaccharides (LPS), widely recognized as endotoxins [32].

However, cyanoprokaryotes can produce also some different from “standard” cyanotoxins alkaloids (e.g., nostodione—Nd) and specific cyanopeptides (CNPs), including NRPs, polyketides (PKs) and their hybrids (NRPs/PKs) [59,60]. Some of them are common in different cyanoprokaryote genera (e.g., anabaenopeptins—Aps, cyanopeptolins—CPs), while others are specific to a single genus (e.g., oscillatorin in *Planktothrix* (*Oscillatoria*) [61] and cryptophycins—Crs in *Nostoc*) [59]. Cyanoprokaryotes are also associated with production of geosmin (Geo) and 2-methylisoborneol (MIB), routinely classified among algal taste and odour compounds (TOC), which are considered non-toxic, but are harmful to humans, animals and ecosystems [32,62,63]. Due to the problems which they cause [32,63] and specifically to the high economic impact of Geo [62], they were included in our analysis.

In marine environments eukaryotic algae of different taxonomic groups, but mainly dinoflagellates from division Pyrrhophyta (Dinophyta), produce a series of toxins which are divided according to the syndrome associated with the exposure to them: diarrhetic (diarrheic) shellfish poisoning toxins—DSP toxins, or dinophysistoxins (okadaic acid—OA, pectenotoxins—PTX and yessotoxins—YTX), paralytic shellfish poisoning toxins—PSP toxins (SXTs), neurotoxic shellfish poisoning toxins—NSP toxins (brevetoxins—Pbtx), ciguatera fish poisoning toxins—CFP toxins (ciguatoxins, maitotoxins) and pfiesteria toxin, a causative agent of with the so-called Estuary-Associated Syndrome [31,64]. Other phycotoxins in marine environments, produced by dinoflagellates, macrophytic red and green algae, diatoms and haptophytes are the amnesic shellfish poisoning toxins—ASP toxins (domoic acid—DA and its isomers), azaspiracids, spirolides, palitoxins (PLTXs), gymnodine, cooliatoxin, zooxanthellatoxins, pinnatoxins, carcharotoxins, caulerpin and caulerpicine, prymnesin, neurymenolide A, etc., classified also chemically by their structure or according to solubility in water and organic solvents [31]. Of note, despite the ever-growing knowledge, at present, the proportion of known versus unknown phycotoxins is not clear [65]. Details of chemical structures, biosynthetic gene clusters, modes of action, associated medical syndromes, spread in aquatic algae and quantitative data on phycotoxins are accessible in a bulk of publications (e.g., [31,32,46,58,59,63,64,66,67,68,69,70,71,72,73,74,75,76,77,78,79] among the many others). They are the basic source of information on toxins of different genera for the provided table, which presents a summary on toxin-producers from the ecological groups discussed in the review. Genus was chosen as the basic taxonomic unit because in most of the publications only strain numbers are used, or authors stress the lack of accurate identifications. When possible, in the text of the review, the recently accepted taxonomic name is provided with old name kept in brackets for easier checking by the reader.

## 3. Phycotoxins—Origin and Possible Biological Role

Given the fact that cyanoprokaryotes are the earliest photosynthetic organisms on the globe and that most toxins are nitrogen-rich, it is possible that the energy and resource demanding toxin production occurs over long evolutionary periods. Some phylogenetic studies demonstrated that phycotoxins such as MCs and NODs, appeared more than 2100 million years ago [46,80,81,82,83], and later the ancient origin of Geo gene was also suggested [62]. It was speculated that the biosynthesis of the remarkable SXTs which occur in two domains of life, Eubacteria and Eukarya, evolved in ancestral cyanobacterium that successfully acquired genes through horizontal gene transfer (HGT), followed by multiple HGT to dinoflagellates, gene losses and rearrangements [46,84]. Within this “ancient origin” belief, the recent sporadic distribution of toxic strains and their co-occurrence with non-toxic ones is explained by inactivation, point mutation or by gene losses [46,62,80,81,85,86,87,88], or alternatively, by frequent HGTs [46,89,90,91,92,93,94].

Considering the reported timescales, it seems very likely that toxic compounds can provide some ecological advantage and have important biological functions in their producers [83,88,95,96]. Since algal toxins affect numerous aquatic and terrestrial organisms, the idea of the primary protective role of phycotoxins was launched years ago [31,51,97]. It uses the fact that algae are the basis of the food web and therefore they have developed toxic compounds to increase their surveillance chance against grazing by herbivores and strong competitive pressure. However, a lot of contradictory evidence against the sole or predominant protective role of phycotoxins was accumulated. Serious doubts come from considering the fact that cyanoprokaryotes are the most ancient phototrophs, which predate the metazoan lineage [83,98,99,100], and therefore, it is more likely that production of phycotoxins may be a response to specific environmental abiotic stressors [100,101]. For example, experiments on the influence of trace metals showed that the presence of toxin appears to give an advantage to MC-producers in the early stages of exposure to severe iron stress and may protect the cell from reactive oxygen species-induced damage [102]. Doubtless, the processes of adaptation can be highly dynamic and strain-specific. Currently, a transcriptomic study of filamentous cyanoprokaryote *Nostoc punctiforme* demonstrated that it can keep low amounts of its metabolites, but its cells are specialized in production of different compounds, which ensures the reduction of overall costs at community level [103]. Therefore, it was supposed that the multicellular algae are better adapted to react fast in changing environmental conditions and that the secondary metabolites can be considered as a type of phenotypic plasticity [103]. The evolutionary role of Geo in niche adaptation was supposed because of its similarity in aquatic and terrestrial strains of cyanoprokaryotes [62]. Some additional arguments, linked with aeroterrestrial and extreme modes of life, used the Snowball Earth hypothesis, according to which, about 600 million years ago the whole Planet was covered by thick ice layer and photosynthetic cyanoprokaryotes survived in mats similar to those, which are recently spread in high alpine or polar regions [104]. In these mats, regarded as small-scale refuges (microrefugia), the grazing pressure is low, which makes toxin production for resistance or allelopathy very unlikely [47,51,97,105,106]. Among the strongest arguments against toxins as grazing defending tool is also the fact that most organisms which produce them have other efficient and cost-effective protections like mucilage sheaths of colloidal polysaccharides and formation of larger colonies [83].

These data and the supposed ancient origin imply that phycotoxins and cyanotoxins in particular have other ecological role(s) is currently emerging. Then, a number of various physiological roles have been speculated and put forward [31,83]. All these hypotheses, unified as “physiological aide”, consider the contribution of phycotoxins in improving the cell physiology in terms of homeostasis, growth rates, antioxidative stress (with which most aeroterrestrial and extremophilic algae have to contend), cell signaling processes, nutrient uptake, detoxication of metal storage (Zn and Cd), iron scavenging and photosynthetic efficiency [31,83,88,107]. Relatively recent the specific AEG isomer of BMAA was discovered in cyanoprokaryotes and was considered as an “echo from pre-RNA world”, a backbone for peptide nucleic acid, which may have been the first genetic molecules for life on earth [108]. The presence of AEG in recent cyanoprokaryotes suggests that the ability for its production is highly conserved, and it still can have some genetic functions [109]. Additionally, some ideas on possible function of phycotoxins as sexual attractants (pheromones) or role in gene regulation, chromosome organization, intraspecific regulation, cell differentiation in colony formations and in shaping the community composition through inter- and intra-species communication were suggested [31,97,100,107,110,111,112,113,114]. However, the strain-specific and diverse laboratory and field results obtained on the physiological aide have made it difficult to generalize [88]. For example, most research was carried on MCs, and it was proved that MC production is strain specific, with up to twenty congeners potentially produced by a single strain, but the exact role of different toxin variants for their producing cells is not fully understood [97,115].

Both main views on the phycotoxin functions—protective role or physiological aide—also lead to the logical but yet unanswered question why toxic and non-toxic strains live together in the same habitats [97]. Some recent studies provide an interesting view on the potential benefit of non-toxic strains from their co-occurring with the toxic ones based on strong link between apoptosis and toxin production [116]. Furthermore, an important discovery that cyanoprokaryotes can contain both hepatotoxins (MCs and NODs) and their antitoxins (Ncps) was made [117]. It led to a new glimpse for interpretation of bioassays, in which toxigenic extracts did not cause the expected detrimental effect even when they contained the necessary toxin amounts but may provide additional help in elucidation the action of the toxins [117].

## 4. Phycotoxins and Factors That Affect Their Production 

To date, most research has focused on the evaluation of environmental conditions that trigger toxin production and its dynamics, which can be essential for understanding of the toxicity and its predicting [31]. Although the role of environmental factors is not sufficiently understood and the results were widely disputed, many physiological studies demonstrated the influence of light, nutrient availability, temperature, pH and dissolved inorganic carbon (DIC) on enhancing or suppressing the production of different toxic compounds [88,96,97,111,118,119,120,121]. Evidence suggested that generally increased temperatures and light favor the growth of toxin producers and can enhance the toxin production [83,88,122], but at times, as for other factors, conflict results. Interestingly, it was proved experimentally that non-toxic strains of *Microcystis* were better competitors for light than its toxic strains [123]. Regarding the nutrient availability, most research aimed at the role of key nutrients, such as nitrogen (N) and phosphorus (P), because of the worldwide acceptance that eutrophication has a major role in the increase of harmful algal blooms in freshwater, estuarine, and coastal marine ecosystems. A recent study based on meta-analysis approach showed that overall, N-rich toxin content in cyanoprokaryotes and dinoflagellate increased with P limitation, while it tended to decrease with N limitation [124]. Although the paleolimnological data on phycotoxins are quite scarce, similar results were obtained on correlation between N and P and MC content in sediments [99]. The role of trace metals, and especially the importance of zinc and iron-limited conditions for production of cyanotoxins (MCs), was also demonstrated [101,102,125]. It has been hypothesized also that the humic acids in land runoff are responsible for chelating enough iron to induce some blooms [33]. For the growth of brackish and marine algae and the production of the toxins as well, salinity is the critical factor with possible important role of biotin, vitamin B12 and thiamine [31,33]. 

Toxin production can be impacted by the physiological status of algae. For example, BMAA production and storage were supposed to be a function of growth and life cycle in experiments with *Calothrix* sp. and *Nodularia spumigena*, in which the toxin amounts increased by age from initial zero [126]. Composition and dynamics of algal populations and the presence of competitor or predator seem to be involved in the toxin production in water bodies [83]. It has been also hypothesized that the planktic or benthic mode of life can influence the production of toxic compounds. For example, comparative genomics of the genus *Planktothrix* demonstrated that the microviridin (MV) gene cluster occurred in all planktonic strains but, with a single exception, it was absent in the benthic strains [127]. 

The hypotheses of physiological aide of toxins in relation with cell signaling were connected with experiments on cultivation conditions for studying the effects regulated by quorum sensing [120]. Although some contrasting results were reported, the impact of high cell density (HD) cultivation on the production of secondary metabolites in two terrestrial *Nostoc* strains revealed the importance of cell density and cell-type specific expression [103,107]. Cultivation under HD conditions optimized the synthesis of CNPs in *N. punctiforme* PCC73102 [103]. In the same way, in conventional cultures of the same strain and symbiotic strain KVJ2, isolated from *Blasia pusilla*, ANBs were in low amounts, while in HD cultures, their peaks were detected [107]. The study allows to suppose that HD conditions are similar to the natural growth of terrestrial cyanoprokaryotes in crusts [107]. This seems to be very different from the situation in aquatic environments where most toxins are diluted and occur in low concentrations, and only during blooms, when the cell density is high, the concentration of signaling molecules in the environment increased significantly, creating a favorable environment for quorum sensing [128]. 

From the analysis of literature, it is possible to conclude that most data were obtained on aquatic toxin-producers and that there is not a big change in the situation with experimental studies on extremophilic algae, as it was seen by Cirés et al. [49], who supposed that this could be explained with the scarcity of toxic cultures from more extreme habitats [49]. Moreover, according to the review of these authors, the production of cyanotoxins can drop off suddenly or stop at conditions close to the environmental extremes, at the border of the growth rate [49].

## 5. Phycotoxins of Aero-Terrestrial, Airborne and Extremophilic Algae

### 5.1. Aeroterrestrial Algae

The broad aeroterrestrial ecological group comprises algae on all types of solid subaerial surfaces (natural or man-made), as well as algae from soil depths, soil fissures and surface crusts in all climatic regions of the world, including polar regions, high alpine areas and hot deserts [15,129]. They have to stand the strong insolation and UV-radiation, desiccation, rapid temperature changes (seasonal and diurnal), low nutrient content and are moistened by percolating water [4].

#### 5.1.1. Phycotoxins of Aeroterrestrial Algae from Ambient Habitats and Contamination of Soils and Plants

##### Phycotoxins of Aeroterrestrial Algae from Ambient Habitats

The biodiversity of aeroterrestrial algae is heterogenous in given localities and habitats, but always two taxonomic groups are outstanding—Cyanoprokaryota and green algae from division Chlorophyta [14,17,25,129]. Although cyanoprokaryotes are significant constituents of subaerial communities, and the first evidence for production of ectocrines by algae came from a culture of aeroterrestrial *Nostoc punctiforme* [130,131], yet there are very few studies which demonstrate toxin production from aeroterrestrial algae in comparison with their aquatic counterparts. The generally low number of studies on this ecological group is a well-known problem, explainable by the need for their proper transportation, followed by time- and effort consuming but obligatory cultivation [132]. All reported data on toxic algae concerned cyanoprokaryotes, and therefore, below, only the Latin names are given, without noting the taxonomic group.

In the last two decades of the 20th century, the first “standard” cyanotoxin, MC-LA, was identified in *Hapalosiphon hibernicus* from Hawaiian soil [66] and different cytotoxic compounds were reported from several common aeroterrestrial genera. The specific nucleoside tubercidin (Tb) was identified as the major metabolite of *Tolypothrix byssoidea* [133]. Previously, Tb was known only from the bacteria *Streptomyces tubercidus* [134]*,* and almost twenty years later, it was found to be the also major metabolite in the marine sponge *Caulospongia biflabellata* [135]*.* Tb is a potent antibiotic and a cytotoxin with antitumor properties, which inhibits DNA, RNA and protein synthesis [135]. In the same period, other aeroterrestrial algae pointed as cytotoxin producers were *Anabaena laxa* [136], *Schizothrix* sp. [137], *Microchaete loktakensis* [138], *Westiellopsis prolifica* [66] and *Nostoc* 31 [139] (Table 1). The research on the non-ribosomal cyclic hexapeptide nostocyclamide, isolated from *Nostoc* 31 [139], continued ([140,141], etc.) and not long ago this cytotoxin was found also in the halotolerant planktonic *Aphanothece halophila* from Dead Sea [142].

Series of works in the 90s of the 20th century led to isolation and identification from the terrestrial *Nostoc* sp. ATCC53789 (lichen symbiont from Arron Island, Scotland) and *Nostoc* GSV224 (ATCC55483, from India) of the novel depsipeptide, cryptophycin (Cr) in a spectrum of more than 25 variants, similar for both strains [143,144,145,146,147]. Further studies proved that Cr biosynthetic gene clusters in both *Nostoc* strains were identical [148] and that arenastatin A, isolated from the marine sponge *Dysidea arenaria* [149], was identical with Cr-1,-3,-4,-24 [147,150]. Almost twenty years later, Cr was found (together with Aps and Nps) in another soil strain, *Nostoc* sp. ASN_M from Iran [151]. More recently, the role of Cr, isolated from *Nostoc* sp. BN KY303912, in animal poisoning incident (duck death) in Iran was supposed [152]. To date, more than 28 natural Crs are known [59,144,153] and Crs are considered to be among of the most potent tumor-selective cytotoxins-suppressors of microtubule assemblages and their dynamics, which prevent microtubules from forming correct mitotic spindle, block the cell cycle at metaphase, cause cell-cycle arrest with subsequent cell death and thus exert strong antiproliferative activity [41,138,139,153,154,155,156,157].

Another “mitotic poison”, nostodione A (Nd A), was isolated from a strain of the aeroterrestrial *Nostoc commune* from Okayama City (Japan) [158]. Its unique carbon skeleton was similar to the natural UV screening alkaloid scytonemin [158,159]. The biological function of Nd A for its producing cells was not elucidated, but its first total synthesis was achieved [160]. Here, we would like to stress that another microtubule spindle targeting (“anti-mitotic”) toxin, neurymenolide A, similar to Cr and Nd A, was found in marine eukaryotic red algae *Neurymenia fraxinifolia* and *Phacelocarpus neuromenyoides* [74,161].

A novel oxazole peptide alkaloid with antibacterial activity, the cytotoxic muscoride A, was isolated from terrestrial and freshwater *Nostoc muscorum* [162].

*Nostoc* is also the sole producer of some weak cytotoxins, which have been declared potent antitoxins, able to block the transport of “standard” cyanotoxins MCs and NODs to hepatocytes: nostocyclopeptides (Ncps), nostopeptolids (Nos), nostoweipeptins (W1-W7) and nostophycin (Table 1), from which the last two were isolated only from aquatic strains of the genus [59,163,164].

Ncps are a small class of naturally produced low molecular NRPs with unique imino linkage in the macrocyclic ring, which do not bind to protein phosphatase (PP) 2A and block the hepatotoxic actions of MCs (MC-LR) and NODs through inhibition the organic anion transporters, OATP1B3 and OATP1B1 (which also mediate the hepatic transport of hormones, drugs, etc.) [59,117,165,166]. Thus, Ncps are potent antitoxins that can serve as antidotes, which efficiently counteract the MC and NOD induced apoptosis and can provide new therapeutic tools to replace toxic and less specific hepatic transport inhibitors [117,166]. Ncps were firstly isolated in two variants (Ncp-A1 and Ncp-A2) from the *Nostoc* strain ATCC5389 from the Aaron Island (Scotland), reported earlier as a Cr-producer [144,165]. More recently, two new Ncps producers were purified from the Baltic Sea [117,167]: *Nostoc*, isolated from a gastropod symbiont, strain XSPORK 13A, which formed different Ncps variant Ncp-M1 (and later was found to form also seven variants of nostoweipeptins, W1-W7 [164]) [117] and a free-living *N. edaphicum* CCNP1411 [167]. The last one, besides the known peptides, Ncp-A1 and Ncp-A2, contained six other compounds (Ncp-E1-L and E2-L, Ncp-E3 and Ncp-E4-L) putatively defined as new Ncp analogues [167].

Nos are cyclic hybrid NRPs/PKs natural products [59], found in different variants from aeroterrestrial *Nostoc* strains: Nos A1-A3 were isolated from the free-living *Nostoc* sp. GSV224 [168,169] and Nos L1-L4—from the symbiotic *Nostoc* sp. UK2almI [164]. During a genome-based study, in which *Nostoc punctiforme* PCC73102 was taken as a model strain capable of facultative entering in symbiotic relations, Nos A and Nos 1052 were found together with Aps (nostamide A and Ap NZ857) and highly unusual MV types N3-N9 [103].

Cyanovirin-N (CV-N) is a low molecular lectin with potent activity against different enveloped viruses but is not toxic for humans (when applied as 0.5–2% gel) [59]. It was firstly isolated from a crude extract of cultured *Nostoc ellipsosporum* from Hawai [59,170]. This 11-kDa polypeptide, identified in a search against HIV, with a clarified mode of action, is of considerable interest in designing drugs against AIDS, but up-to-now the physiological role of this compound for its producer is not known [59,170,171,172,173].

Nostosins (Ns) are linear NRPs firstly isolated and characterized from a terrestrial *Nostoc* sp. FSN from a paddy field in Golestan Province (Iran) [174]. These low molecular linear tripeptides, which are the smallest and most potent trypsin inhibitors, exist in six variants (Ns A-F) [59,174,175]. Although found only in *Nostoc*, Ns have some similarity in the effects with the linear CNPs aeruginosins (Aers, namely, Aer-GH553, Aer-EI461, Aer-298B) and spumigin (spumigin E in particular), known from other cyanoprokaryotes [59,174]. Aers, in particular, were also found in aeroterrestrial *Nostoc* with Aer-865 being the first variant, discovered in the strain Lukešová 30/93, isolated from a mountain forest soil in the Czech Republic [176]. This novel tetrapeptide has unique structure, not typical for other Aers, such as the presence of uronic acid and a fatty acid moiety, and therefore it was supposed to be a type of evolutionary intermediate [176]. Moreover, the finding of this new variant with strong anti-inflammatory activity and almost no cytotoxicity suggested discovery of novel potential immunomodulating agent [176]. Other protease inhibitors, the novel peptides banyasides, containing unique amino acids, were isolated from a freshwater bloom of *Nostoc* in three variants (A1-A2 and B3) [177]. Later, these glycopeptides, similar to Aers, were identified together with variants from other peptides including nostoginins, microginins and Aps in 45 from 133 terrestrial *Nostoc* and *Nostoc*-like strains, isolated from various alkali grassland habitats in Great Hungarian Plain, in which MCs were not found [178]. Aps, protein inhibitors, which are produced also by other cyanoprokaryotes, were found together with Cr and Ncps in a *Nostoc* strain ASN_M collected from a paddy field in Iran [151] and their novel variant, Ap NZ857, was characterized during integrated transcriptional and metabolomics studies with HD cultivation of the terrestrial *Nostoc punctiforme* strain PCC 73,102 [103,107].

In this regard, we have to outline, that *Nostoc* can produce also other protein inhibitors, CPs [179], commonly detected in other cyanoprokaryotes [180], but similarly to Aps and microginins, these NRPs frequently have some unique structure modifications [59]. For example, in *Nostoc*, the oligopeptide compounds with similar to CPs structures are known as nostopeptins, insulapeptolides and nostocyclins, isolated mostly from versatile aquatic environments (Table 1) [59].

Besides all mentioned above NRPs, *Nostoc* can produce some ribosomal peptides, which inhibit proteases and elastase and have been found also in other cyanoprokaryotes [59,120]. Such example for ribosomally synthesized and post-translationally modified peptides (RiPPs) is the unique 16-membered family of the toxins microviridins (MVs), named after their first discovered producer—the aquatic toxic *Microcystis viridis* (strain NIES-102) [181,182]. Later, more variants and MVs were found in *Microcystis* and in other bloom-forming species from the genera *Planktothrix*, *Anabaena* and *Nodularia* and in more structural variants in other bacterial groups [120,183,184,185,186,187,188,189,190]. In aeroterrestrial algae, MVs were reported from *Nostoc minutum* NIES-26 [191] and *N. punctiform*e PCC73102, in which Nos and Aps were also found [103]. Although these oligopeptides are largely studied, their low yield is considered one of the bottlenecks for their production and evaluation in cyanoprokaryotes [120].

*Nostoc* was reported also as producer of “standard” cyanotoxins. *Nostoc* is a rare example of non-*Nodularia* producer of the cyclic pentapeptide NOD, but its production by *Nostoc* was at 100-fold lower levels than that observed for the aquatic, NOD-producing *Nodularia* sp. (Table 1) [59,192,193]. The synthesis of NOD and [L-Har2]-Nod by two terrestrial cycad symbiont isolates of *Nostoc* sp. ‘*Macrozamia serpentina* 73.1’ and *Nostoc* sp. ‘*Macrozamia riedlei* 65.1’ was confirmed genetically and chemically [192]. In *Nostoc* symbionts, which inhabit the roots of *Cycas* and *Macrozamia*, BMAA was identified [126,194,195], but not long ago, it was isolated also from four free-living marine, brackish and freshwater *Nostoc* strains [126,196]. MCs were found in few free-living strains (some of which from aquatic habitats) [47,197,198,199,200,201,202,203,204,205,206,207,208] and in some symbiotic strains, isolated from terrestrial lichens, liverworts and flowering plants [126,209,210]. SXTs were reported from *Nostoc microscopicum*, *N. linkia* and *N. punctiforme* [200,201] and peaks with retention time near to the ATX were detected by HPLC in *N. linckia* [200]. Interestingly, experiments with *Nostoc flagelliforme*, which is commonly used for food in China, did not show adverse effects on rats, which may indicate its safety for human consumption [211].

*Nostoc* was pointed also as the main causative agent for the production of Geo in soils [62]. This compound, due to its specific earthy and musty taste, is better studied from freshwaters, but its release has been associated also with the terrestrial cyanoprokaryotes *Aphanizomenon*, *Coelosphaerium*, *Dolichospermum*, *Phormidium* and *Tychonema*. Recently, a new aeroterrestrial species, producer of Geo, was described from the Lisbon city center – *Microcoleus asticus* [62].

Other newly described terrestrial cyanoprokaryotes, isolated from dried paddy fields of Mazandaran (Iran), *Neowestiellopsis*, with two species *N. persica* and *N. bilateralis* were supposed to be cryptic taxa of true-branching cyanoprokaryotes [212]. Considering that the phylogenetically close *Westiellopsis prolifica* has been shown as producer of toxic antibacterial westiellamide (a bistratamide-related cyclic peptide) in soils and MCs in freshwaters [66,213], it is possible to speculate similar toxigenic abilities in the aeroterrestrial *Neowestiellopsis*.

In a search for neurotoxins among different ecological and morphological cyanoprokaryotes, BMAA production was proved in strains of the aeroterrestrial genera *Chlorogloeopsis* (isolated from Indian soil); *Chroococcidiopsis* (isolated from marine rock in India) and *Calothrix*, *Phormidium* and *Plectonema* (with unknown origin) [126] (Table 1).

The ubiquity of soil and aerophytic algae explains their importance for wide-human exposure [126]. Therefore, the interest in aeroterrestrial algae and their potential toxicity is increasing due to increasing safe requirements and new eco-friendly processes, related with the application of algae as soil conditioners [214] and in different cosmetic products [24].

##### Phycotoxins Contamination of Soils and Plants

Presence of phycotoxins in soil algae can be a potential source for soil contamination, but its main cause is considered to be the irrigation by infested water. Water used for irrigation, comes from natural or man-made waterbodies but commonly is not a subject for special quality control, and on the background of the globally increasing frequency of algal blooms in freshwaters, the concern from soil contamination by phycotoxins is emerging [115]. Two additional sources are the removed amounts of algal biomass from waterbodies to agricultural lands or forests, without any additional treatment and the direct fertilization of soils by cyanoprokaryotes, experienced in some countries [115]. The risk associated with toxin stocks for further contamination of groundwaters through infiltration from soils and phycotoxin migration which follows precipitation was already shown by different studies [115].

Therefore, starting from the last decade of 20th century, the research on toxin accumulation and influence on the commercially cultivated plants after irrigation by contaminated water became a specific aspect of the studies of phycotoxins in soils (e.g., [34,215,216,217,218,219,220,221,222,223,224,225,226,227]. Although constantly increasing, the number of experiments is limited with exceptional work in natural conditions, and few studies addressed the plants susceptibility to phycotoxins at environmentally relevant concentrations [225,226,228]. Despite some crop plants and some aquatic macrophytes (e.g., *Ceratophyllum demersum*, *Elodea canadensis*, *Phragmites australis*, *Vesicularia dubyana*, and *Azolla filiculoides*) having demonstrated possibilities for self-decontamination, for development of tolerance or resistance to cyanotoxins and even registered better growth at certain MC or CYN concentrations, there is a general consensus on the strong negative economic impact with health risk hazards of toxin-containing water, applied as standard or spray irrigation [227,228,229,230,231,232,233,234,235,236,237,238,239,240]. Moreover, after conventional treatment, water still can contain extracellular phycotoxins (e.g., MCs), released after lysis of cells [236]. All studies showed that aquatic macrophytes absorb through all submerged parts [229,231], and crop plants irrigated by infested water can absorb phycotoxins through roots or accumulate it on leaves, and that the effects can be harmful for humans even in cases of no visible changes [115,225,241]. For example, cyanotoxins can affect the plants metabolism, which can lead to the accumulation of potential allergenic proteins [242]. Therefore, the consumption of contaminated vegetables and food supplements became recognized as additional important exposure route [228,236,243].

According to the accumulated knowledge, such infested water may impair the photosynthesis, inflict severe oxidative stress, affect mineral accumulation by plants, provoke adverse histological modifications in primary plant tissues, reduce the germination rate of seeds, inhibit the growth and development of seedlings, cause alteration of the quality and the productivity of crop plants with a potential to move these toxins into farm animals and thus have detrimental impact in the whole food chain [115,217,223,226,227,228,236,244,245,246]. The reported effects on metabolic processes and related germination and growth rates depend both on the toxin variant used and on the detoxication enzymatic activity of tested plants, such as *Azolla filiculoides*, *Anethum graveolens*, *Brassica napus*, *Brassica cretica* (Syn.: *Brassica oleracea* var. *italica*), *Ceratophyllum demersum*, *Cicer arietinum*, *Coriandrum sativum*, *Cucumis sativus*, *Daucus carota*, *Elodea canadensis*, *Eruca vesicaria* (Syn.: *Eruca sativa*), *Glycine max*, *Lactuca sativa*, *Lens culinaris* (Syn.: *Lens esculenta*), *Lepidium sativum*, *Lolium perenne*, *Medicago sativa*, *Oryza sativa*, *Petroselinum crispum*, *Phaseolus vulgaris*, *Phragmites australis*, *Pisum sativum*, *Raphanus* sp., *Sinapis alba*, *Spinacia oleracea*, *Solanum lycopersicum*, *Solanum tuberosum*, *Trifolium repens*, *Triticum durum*, *Vesicularia dubyana*, *Vicia faba*, *Vigna radiata*, *Zea mays*, etc. [34,223,226,229,230,233,238,239,247]. For example, *Pisum sativum* (pea) was one of the most sensitive to MC-LR exposure plants contrasting to the more resistant *Lens culinaris* (lentil) [223]. After exposure to MCs visible changes in *Cucumis sativus* (cucumber) and *Spinacia oleracea* (spinach) were observed [228,248], while no changes were found in *Lactuca sativa* (lettuce) and in *Vicia faba* (faba bean) [249,250]. Experiments under varying conditions demonstrated the significant CYN uptake by *Brassica cretica* (Starbor, kale), *Brassica juncea* (brown mustard) and *Sinapsis alba* (white mustard) with accumulation in leaves and roots [251]. *Sinapsis alba* and *Oryza sativa* (Asian rice) can be affected by CYN with visible anatomy modifications and growth inhibition, with possible tissue necrosis concomitant with oxidative stress caused by longer exposures [115,252,253]. In contrast, the experiments with subchronic exposure to CYN by the aquatic fern *Azolla filiculoides* demonstrated the low uptake of CYN and no effect on photosynthesis and protein synthesis, which pointed towards to its safe use as biofertilizer and as food and forage source but also indicated that *Azolla* is not suitable for CYN phytoremediation [240]. The marine phycotoxin OA can efficiently block chlorophyll accumulation in *Zea mays* (maize) leaves [254], as well as the root hair growth and can alter the cortical cell shape in *Arabidopsis thaliana* [255]. OA in combination with MC-LR or MC-LR alone act as protein phosphatase inhibitors and inducers of reactive oxygen species production and can cause versatile genetic and physiological changes in different plants like *Ipomoea batatas* (sweet potatoes), *Spinacia oleracea*, *Lepidium sativum* (cress, watercress) seedlings, *Solanum lycopersicum* (tomato) and *Oryza sativa* (for details see [217]).

As it could be seen, up-to-now, studies addressed mostly MCs due to the long-term accumulated evidence on their broad prevalence in eutrophic waters, while few data are available on dermatotoxins and neurotoxins [65,115,237,240]. MCs, due to their ring structure, are stable under sunlight, and their long persistence in soils, with half-life between 6 and 17.8 days, was proved [220,236,256,257]. Similarly, MCs introduced through irrigation water are bioavailable to crop systems during long period but can be affected also by soil structure, chemical composition and local or modified microbiota [218,221,227,235,246,258,259,260,261,262,263,264,265,266]. To exemplify, sandy soil was incapable to remove cyanotoxins [259] but modified clays and soils can accelerate the improvement of the quality in eco-friendly manner [265,267,268]. The degradation and removal of MCs from the soils in agroecosystems or decrease of plant stress seems to be possible due to microbial activity in the rhizosphere [246]. The plants cultivated in hydroponic systems accumulated more MCs in their organs in comparison with soil-grown plants [269,270], and this is explainable in cases when the roots were in direct contact with toxin solutions [217]. Specific study on lettuce showed that washing and rinsing is not enough to remove the toxic cells from the leaves [255].

In regard with the movement of MCs through the food chains, the research was oriented towards their absorption, translocation and accumulation in plants, and in edible plants in particular (e.g., [34,217,226,232,235,239,241,246,248,255,270]). Additional problem comes from the fact that plants can absorb MC-LR in low external concentrations (0.5 µg L^−1^); this was firstly shown on aquatic macrophytes [229,231]. Since toxin accumulation in edible plants could pose a potential risk for human and animal health, if the MC intake exceeded the recommended tolerable limits, most studies addressed MC-LR for which WHO limits exist [226]. For example, accumulation of MCs in concentrations over the threshold tolerable daily intake (TDI) 0.04 µg kg^−1^ body weight, announced by WHO in 2004 (and confirmed in 2020 [271]), for humans was reported for cucumber, carrot and lettuce [34,222,232,271] and recalculated for clover grazed by cattle [222]. Currently, more provisional health-based guideline values were announced: for short-term (ST) and lifetime (LT) drinking water (DW) and recreational water (RW) for MC-LR (ST ≈ 12 μg L^−1^ and LT ≈ 1 μg L^−1^ in DW, ≈24 μg L^−1^ in RW), for ATX (ST ≈ 30 μg L^−1^ in DW and ≈60 μg L^−1^ in RW) and for CYN (LT ≈ 0.7 μg L^−1^ and ST 3 μg L^−1^ in DW), as well as for SXTs acute guideline value for DW ≈ 3 μg L^−1^ [271,272,273,274].

Despite all findings cited above, more studies are needed for full understanding of all factors which influence the toxin uptake and accumulation, differences in absorption by different crop species and their exposure organs, all possible morphological and physiological effects on plants and on toxin persistence after harvesting [34]. Doubtless, all accumulated evidence confirmed the earliest suggestions that phycotoxin uptake by edible plants could be detrimental for human health [255,275]. This raised concerns about food safety, showing that crop production environments have to be free of phycotoxins at least during the cultivation period, and the relevant monitoring in both aquatic and land habitats of the agricultural systems and plants affected by cyanotoxin contamination is needed [34,239].

#### 5.1.2. Aeroterrestrial Endolithic Algae

Endolithic algae inhabit the inner surfaces or fissures of stones and rocks and comprise a very specific community, which after the pioneer works by Imre Friedmann and his colleagues (e.g., [276,277,278,279] is considered extremophilic [7,280,281]. Nevertheless, little is known about the global biodiversity of algal endoliths, but despite some fine-scale partitioning depending on the rock types and local climate conditions, the similarity in species composition allows to generalize that cyanoprokaryotes, and the polyextremophilic BMAA producing [126] genus *Chroococcidiopsis* in particular, often dominates [4,13,280,281,282,283,284,285,286,287]. Among all recorded endolithic algae, occur other genera, from which toxigenic species have been found in different, mainly aquatic habitats: *Anabaena*, *Aphanocapsa*, *Lyngbya*, *Phormidium* and *Plectonema* (e.g., [276,277,278,279,284,287,288,289,290,291,292,293,294,295]) (Table 1).

Despite of ever-growing number of papers dedicated to the microorganisms in the rocks, there is a single record of phycotoxins from the endolithic strain of *Pseudocapsa dubia*, isolated from a marbel building situated at 353 m a.s.l. in Spain, in which MC-RR and MC-YR were detected [50]. Currently, a number of secondary metabolites, produced by endolithic cyanoprokaryotes, was found and was supposed to be possibly involved in the competition with eukaryotic algae (e.g., green *Trebouxia*) for access to the limited space suitable for photosynthesis within the ignimbrite rocks [296]. Some of these secondary metabolites were with potent antimicrobial activity, including NRPs and PKs, a number of genes for which were detected [296].

#### 5.1.3. Aeroterrestrial Algae from Deserts and Polar Regions

Deserts occupy approximately 15% of the Earth surface and are spread in all geographical realms [7]. On the basis of precipitation/evaporation ratios they are classified as subtropical, cool coastal, cold winter and polar [7]. Algae in deserts have to survive, grow and reproduce in even more harsh arid or semiarid environmental conditions in comparison with other aeroterrestrial algae. Their adaptive traits allow the organisms to thrive both balances of thermal and water energy [25], but often they experience high salinity in addition to strong insolation, temperature fluctuations and low nutrient supply [7].

Data on aeroterrestrial algae from the hot deserts of the world addressed mainly soil and different lithophytic communities (for references, see [297,298]). Despite some dissimilarities, caused by soil and rock types and specific local conditions, cyanoprokaryotes were the most important inhabitants of these arid environments [297]. The significance of their exopolysaccharides to enhance soil stability and water retention, as well as for architecture of biological crusts and providing of microhabitats, was well recognized [299,300,301]. Comparison of species composition showed that 72% of the algae found in the lithophytic communities of Negev have been recorded in other deserts from Africa, America and Asia, with *Nostoc commune*, *N. punctiforme*, *Nodularia harveyana*, *Schizothrix arenaria*, *S. fresii*, *S*, *lenormandiana*, *Synechococcus elongatus*, *Tolypothrix byssoidea* being the most widespread [297]. Similarly, *Nostoc*, *Coleofasciculus (*Syn. *Microcoleus* p.p.) and *Phormidium* s.l. can be outlined as common in desert soils [281,298,301,302]. Currently, a close relative of *Coleofasciculus chthonoplastes (*Syn. *Microcoleus chtonoplastes*) from hot deserts soils was described as a new genus, *Desertifilum* [297]. The transect study covering 800 km and three soil types (lateritic soils, red soils and soils burdened by mine-waste) in arid region in eastern India revealed the general common composition of the biological crusts, which comprised *Scytonema*, *Tolypothrix* and *Lyngbya* along with few species from *Cylindrospermum*, *Nostoc*, *Calothrix* and *Fischerella* [303]. Although most of the abovementioned species were toxigenic in other habitats (Table 1), algae from hot deserts and other arid habitats have been rarely studied in relation to phycotoxins.

However, even the few conducted studies allowed to suggest the associated health risk. It was supposed that the inhalation of dust containing dried, suspended cyanobacteria during dust storms, may be an important exposure route that adversely impacts human health in desert regions [304]. Earlier, a causative link between promoting or enhancing amyothrophic lateral sclerosis (ALS) of Gulf War veterans and chronic exposure to low amounts of BMAA from cyanoprokaryotes in desert dust was supposed [305]. In algal soil crusts from hot deserts, dry riverbeds and supertidal salt flats in Qatar MCs and BMAA with its isomers DAB, BAMA and AEG were found, and GTX was detected by enzyme inhibition assay analysis [109,304,305]. BMAA and DAB were already known as neurotoxins, but for the AEG, isolated from Qatar material, for the first time, preliminary toxicity assays with the nauplii of *Artemia salina* were performed and indicated its low toxicity [109]. However, it was supposed that some synergistic or additive effects of common exposure to BMAA, AEG and DAB could occur increasing the risk of adverse health effects [109]. The most important constituent of desert soil crusts from all environments in Qatar was *Microcoleus*, and it was presumed as primary MC producer, but since it was occasionally intermingled with filaments of *Phormidium*, it was considered to be the second potential candidate, while for BMAA a range of possible producers was speculated [304,305]. Later, adverse effects on dogs in the region were supposed to be primarily caused by ATX [306].

Algae are well-known also as first colonizers of the pyroclastic substrates formed after volcano eruptions and of hydrothermal deposits formed around hot springs in volcanic geothermal regions, which are often iron poor but silica rich [7]. Cyanoprokaryotes were found as significant participants in such communities both on the substrate surfaces and inside the rocks [293,307,308]. In these works, species from genera *Anabaena*, *Leptolyngbya*, *Lyngbya*, *Oscillatoria* and *Phormidium* have been recorded by conventional light microscopy, from which toxic species are known (Table 1). Genetic studies indicated that much of the diversity on these new landforms were either closely related to uncultured organisms or distinct from any reported 16S rRNA gene sequences. The presence of cyanoprokaryotes was detected by 16S rRNA in vegetated volcano soils of Mount St Helens and around Kilauea volcano at Hawai [309,310], but their toxins have not been investigated.

In the warmer northerly latitudes of Antarctic Peninsula and on the Signy and Marion islands, the so-called fellfields with thaw-freeze regime and soils of high porosity are widely spread, which are covered by algal mats in their uppermost layer of 1 mm [311,312]. These mats comprise of different algae but generally are dominated by non-heterocytous filamentous cyanoprokaryotes *Phormidium autumnale* and *Pseudanabena catenate*, sometimes intermingled with diatoms like *Pinnularia borealis* [7]. The first two species have been reported in freshwaters as toxigenic (Table 1).

In the Dry Valleys of Antarctica, the coldest and driest Earth deserts cover about 4800 km^2^; cyanoprokaryotes are the most important primary aeroterrestrial soil and epilithic (also hypolithic) phototrophs [7], among which early studies identified species of the genera *Calothrix*, *Nostoc* and *Scytonema* [295] and on the sublithic soils *Plectonema*-like clones were identified [292]. Later, by metagenomic analyses, higher biodiversity from the orders Chroococcales, Oscillatoriales and Nostocales was discovered [313,314]. An interesting result from these studies was that the nearby lakes were the source of species for the terrestrial mats, and therefore, it was supposed that their cyanoprokaryotes could be toxic in the same way like their aquatic counterparts. Studies of MCs in hydroterrestrial Antarctic mats from the Marshall Valley (with *Nostoc* as relevant cyanotoxin producer [47,106]) and from McMurdo Dry Valley identified MC-LR, MC-FR and MC-RR and eight new MC congeners with specific glycine containing modifications [208].

In the Nearctic, LM and 16S rRNA gene analysis of the soil crusts in the Colorado Plateau and in the peculiar mesothermal rain shadow Chihuahuan desert revealed dominance of *Microcoleus vaginatus*, *Nostoc* spp. and *Scytonema* spp. depending on the successional stage [300]. Despite toxin analyses on these samples not being conducted, we have to note that toxic species from these genera have reported (Table 1). In the Arctic, firstly the presence of MC-LR and ATX was demonstrated in biocrusts from the North-West coast of Spitsbergen [315]. This was also the first documentation of ATXs from the Arctic, because MCs were found earlier in Arctic freshwaters [316]. According to the pilot identification of biocrust inhabitants, it was tentatively suggested that *Nostoc paludosum*, *Nostoc* sp. and *Chroococcus* sp. were the possible MC-producers, and *Oscillatoria* and *Phormidium* were related with ATXs production [315].

In the first molecular survey of biological crusts from the Arctic and Antarctic green algal genera, *Chloromonas*, *Coccomyxa*, *Elliptochloris* and cyanoprokaryote *Nostoc* were identified in all samples regardless of origin, thus confirming their ubiquitous distribution, with high abundance of Cyanoprokaryota and frequent occurrence of two diatoms (*Amphora*, *Stauroneis*) [317]. This study did not check toxigenic species, but *Nostoc* and *Amphora* are potentially toxic genera (Table 1).

#### 5.1.4. Aeroterrestrial algae of Hypersaline Environments

Data on the algal biodiversity of hypersaline environments concerned mainly the benthic mats in hypersaline coastal lagoons, saltworks and some inland hypersaline lakes, but even fewer studies addressed the terrestrial algae on their shores or algae of hypersaline soils (e.g., salt flats, evaporite crusts, solonchaks) [318,319,320,321]. Most of them, and the salt flats in particular, experience frequent changes in the salinity, diel and seasonal temperatures and water ability because they are unprotected by the moderating effects of the permanent water [319]. Despite the scanty information, it is possible to state that contrary to the earlier opinions on low biodiversity of these habitats, most studies revealed relatively rich phycoflora in oxygenic layers, especially in regard to cyanoprokaryotes [318,320,321,322]. They dominate or co-dominate with diatoms or green algae in soils and biological crusts in these environments, often representing more than half of the recorded species [319,320,321,322,323,324]. Despite some differences in the detailed species composition and abundance, all studies proved the general prevalence of filamentous non-heterocytous algae (e.g., *Geitlerinema*, *Halomicronema*, *Komphovoron*, *Leptolyngbya*, *Lyngbya*, *Microcoleus*, *Phormidium*, *Pseudanabaena*) in these soils, with some differences in the abundance of the colonial (e.g., *Aphanocapsa*, *Aphanothece*) and filamentous heterocytous (*Anabaena*, *Calothrix*, *Nodularia*, *Nostoc*) algae depending on the localities.

On the salt flat shore of the very peculiar, shallow hypersaline Don Juan Pond (Victoria Land, Antarctica), which contains saturated CaCl_2_ brine, a 2–5 mm thick mat of about 1 ton weight was found to cover about 600 m^2^ [325]. The pilot light microscopic observations revealed that it was formed mainly by entangled filaments of *Oscillatoria-*type, intermingled with *Gloeocapsa* and some green cells of *Chlorella* and *Dunaliella* types and yellow-green unidentified tetrads, with occasional presence of diatoms, suggesting the worth of more detailed studies [325,326].

As it could be seen, the species lists provided from different regions of the world contained cyanoprokaryote taxa, reported as real or potential toxin-producers from other habitats (Table 1). This allows us tentatively to suppose that future aimed measurements can reveal phycotoxins, most probably cyanotoxins, in hypersaline aeroterrestrial environments.

### 5.2. Airborne Toxigenic Algae and Their Toxins

Airborne algae, “the free-floating algae in the atmosphere” [327] (p. 2), are now universally recognized as a component of the naturally occurring aerial biota and comprise a considering part of atmospheric bioaerosols [20,328]. Similar to “standard” aeroterrestrial algae, the airborne organisms have to survive the UV-radiation, desiccation and broad temperature variations [329]. Despite these stressful factors, more than 350 species are known, with the highest diversity of green algae and cyanoprokaryotes, followed by diatoms and yellow-green algae [19,20,327]. It has been commonly accepted that cyanoprokaryotes are most abundant in temperate regions, while green algae dominate in tropics [327,330,331], but these geographic peculiarities can be strongly dependent on the sampling season [332].

The role of airborne algae in inducing respiratory problems and skin reactions has been recognized long ago (e.g., [333]) and investigated in series of works, summarized in subsequent reviews [19,20,327]. In these works, some green algal species, which have never been reported as toxin producers, have been pointed as allergenic components of the aerosols: *Chlamydomonas* sp., *Chlorella* sp., *Coccomyxa dispar*, *Myrmecia* sp., *Neochloris* sp., *Palmella* sp. and *Scenedesmus* spp. [19]. However, they were the least studied organisms in aerosols in terms of hazardous health effects [20,334], and yet there are no WHO standard levels determined for them in air quality guidelines [19,271,272,273,274,327]. Nevertheless, the knowledge on the potential toxin producers among them has been accumulated with increasing accepting inhalation and skin contact as significant exposure routes [19].

Currently, about 20 species identified in aerobiological studies are known as toxin producers in their natural environments. They belong to cyanoprokaryote genera *Anabaena* s.l., *Anabaenopsis*, *Arthrospira*, *Cylindrospermum*, *Gloeocapsa*, *Hapalosiphon*, *Leptolyngbya*, *Lyngbya* s.l., *Microcystis*, *Nodularia*, *Nostoc*, *Oscillatoria* s.l., *Phormidium* s.l., *Pseudanabaena*, *Snowella*, *Synechococcus*, *Synechocystis*, *Westiellopsis* and *Woronichinia*; to the dinoflagellate genera *Gymnodinium* s.l., *Peridinium* s.l., and *Prorocentrum* and to diatom genus *Amphora* [19,20,66,328,335,336] (Table 1). Among the most frequently occurring was *Lyngbya*, which means that it was found in >40% of the sampling areas, in every climatic zone, and therefore, it may pose serious threat to the air quality and human health [19]. Regarding this, we have to note that the analysis of data accumulated since the first publication of Ehrenberg [337] on the materials collected by Charles Darwin [338] revealed that only less than 20% of the airborne algae were common [19], while all others depended on the geographical, topographical, climatic, diurnal and seasonal factors [19,328]. At the same time, locally, some potentially toxigenic and allergenic algae can be common in the air of a given region, as it was well exemplified in a study over the Varanasi city of India, where *Nostoc muscorum* and *Phormidium fragile* were found and outlined as hazardous species [339]. Soon after, *N. muscorum* was proved as MC-producer in freshwaters [205].

Generally, the dispersal distances are mainly short, less than 1 km, as it was shown for one of the best-known toxigenic species—*Microcystis aeruginosa*, transported by the wind from the original blooming pool [340]. The dispersal distance for algae could be even longer depending on meteorological factors such as wind velocity and direction, air humidity and also from the algal resistance to desiccation stress [327,330,338,341]. Doubtless, the diversity and abundance of airborne algae depend on the communities at the source. Considering the increase of harmful algal blooms triggered by global warming and increased nutrient loading, it is possible to suppose that the resuspension in the air from the aquatic habitats will be enhanced, increasing the importance of inhalation as exposure route [19,20]. There is accumulating evidence that recreational activities in water bodies with harmful algal blooms can generate aerosolized toxins. The aerosols can contain diverse toxins from different algal groups depending on the given bloom causative agent [19,20,327].

Most research concerned the toxic aerosols obtained from the red tides of the marine dinoflagellate *Karenia brevis* with the effects of its Pbtxs being the most studied phenomenon due to high deposition efficiency of its small-sized particles (6–10 µm) [342,343,344,345,346,347,348]. Marine aerosols can also contain PLTXs, produced by dinoflagellates from the genus *Ostreopsis* [19,349,350] (Table 1).

Inhalation of aerosolized phycotoxins from freshwaters was believed to be spatially associated with unusual high incidences of sporadic ALS in a population with chronic exposure to frequent lake blooms (mostly of *Anabaena* and *Microcystis*) with BMAA (and high concentrations of MCs in lake sediments) and with the increased respiratory intoxications during *Microcystis aeruginosa* blooms with MC [351,352,353,354,355]. Moreover, during the experiments with animals (mice), the inhaled phycotoxins (MC-LR and ATX) were more effective in lower doses than the ingested toxins, and nasally inhaled MCs were ten times more available than the ingested ones [356,357,358].

Adverse effects of inhalation of dusts, containing particles of soil algae with neurotoxins (BMAA in particular) and possible relation with ALS were reported in the discussed above study of hot desert crusts in Qatar [305]. Airborne cyanoprokaryotes possess also endotoxins LPS, suspected to cause health problems [19].

Airborne algae are permanent constituents not only of outdoor but also of indoor environments [129,327,359]. Although they were largely ignored group in indoor environments, the results from the few conducted studies demonstrated the cyanoprokaryote dominance with representatives of the toxigenic genera *Anabaena*, *Nostoc*, *Phormidium and Schizothrix* [328,359,360,361,362]. Inside buildings human movement seemed to be important dispersal vector for airborne algae and this has to be considered in future targeted studies important because of the concern from “sick building syndrome” declared by WHO [328,363].

Other possible deterioration effects of toxic airborne algae are related with their transport and subsequent possible deposition in water bodies, where they can cause harmful blooms [327,364].

According to data summarized above, it becomes clear that toxigenic airborne algae have origin in other ecological groups and their toxicity is hardly related with the newly occupied atmospheric environment.

### 5.3. Extremophilic Algae and their Phycotoxins

#### 5.3.1. Toxigenic Cave Algae

In the natural cave ecosystems, characterized by scarcity of light, water and nutrients but almost constant microclimate conditions, algae are the main photosynthetic components [4]. The estimations of their total numbers strongly varied (627 [365] and 340 [366]), but the most diverse [up to 60%] and commonly the most abundant cave inhabitants are the cyanoprokaryotes, followed by green algae and diatoms (e.g., [367,368,369,370]). The phycoflora of the artificially illuminated caves (show caves), named lamp flora, although comprising different species, generally exhibit a similar pattern of biodiversity with highest contribution of cyanoprokaryotes, green algae and diatoms [371,372].

According to the revealed general species composition, it is possible to suppose that at least some of the cave cyanoprokaryotes may be toxic since almost all aeroterrestrial and extremophilic toxigenic genera have been found there (Table 1). However, to our best knowledge, there are no studies on toxic algae neither in natural nor in the show caves. The only exceptions are the BMAA-producing *Scytonema* PCC 7110 from a limestone Bermuda cave [126] and the epilithic MC-LY containing strain of *Scytonema drilosiphon*, isolated from a cave, situated at 250 m a.s.l. in Spain [50].

#### 5.3.2. Toxigenic Acidophilic and Toxigenic Peat-Bog Algae

Organisms with optimal growth below pH 3 are classified as acidophiles [13]. They occupy environments such as the outflows of mine drainages, solfataric areas which give off sulphureous gases and steams, fumaroles, acidic hot springs, coal spoils and bioreactors [13]. In these extreme habitats, the biodiversity of algae is low, and the best-known are the obligate acidophilic and moderate thermophilic unicellular red algae from order Cyanidiales, which can thrive pH between 0 and 4 and temperatures up to 57 °C [13,373]. Broader pH range can be tolerated by the green *Chlamydomonas acidophila* (pH 3.8) and the golden flagellate *Ochromonas* sp. LG strain [374]. From all these acidophiles, only *Ochromonas* from aquatic habitats have been documented as a potential toxin producer (Table 1).

The most common acidic aquatic habitats occupied by algae are the peat bogs and mires. Often, they include springs of streams and rivers and therefore provide important drinking water sources. The peat (turf) itself is traditionally used as an energy source but can be applied in gardening, agriculture (incl. greenhouse horticulture), can be used in filter systems and for medical purposes and balneotherapy as well [375]. Therefore, the importance of studies on hazardous algae in peatlands is increasingly important. In classical acidic peat bogs, with pH below 5, cyanoprokaryote genera such as *Aphanothece*, *Chroococcus*, *Hapalosiphon*, *Merismopedia*, *Nostoc* and *Tolypothrix* are commonly known [4]. Despite the presence of these potentially toxigenic genera, there are very few targeted studies on harmful algae and their toxins in acidic peat bogs. The risk from possible massive proliferation of *Microcystis* (*M. aeruginosa*) and the eventual occurrence of potent toxic cyanobacteria blooms was demonstrated in a study of a typical acidic peat bog of in Algeria [376]. Abundant development of *Microcystis* (*M. aeruginosa*, *M. wesenbergii*, *M. grevillea*) was detected in the peculiar mountain swamp Choklyovo Blato, long used as a sanitary peatery in Bulgaria [377]. In an epipelic strain of *Pseudanabaena frigida*, isolated from peat bog situated at 1600 m a.s.l. in Spain, MC-LR was proved [50].

#### 5.3.3. Toxigenic Algae of Hypersaline Environments

Hypersaline environments are widely distributed on our planet, and their inhabitants have to cope not only with high ionic composition but also with alkaline pH values, low oxygen availability, high or low temperatures, presence of heavy metals or other toxic compounds [378]. Based on their ionic composition and origin, hypersaline lakes can be classified either as thalassohaline or athallasohaline [378]. Thalassohaline waterbodies originate from evaporation of sea water and such are most neutral salt lakes and saltwork/salterns, while the alkaline soda lakes are of land origin and are atahalassohaline. In all hypersaline environments, cyanoprokaryotes are among the most diverse inhabitants [31,379].

##### Toxigenic Halophilic Algae

Coastal salterns are man-made ponds constructed by man to produce salt through evaporation of sea water, which include a gradient of salinities from the sea water (35 g L^−1^ Nacl) to halite saturation (>300 g L^−1^) and are frequently experiencing fluctuations in salinity [7,319,380]. Much of the understanding of halophilic organisms comes from studies of these waterbodies. Considering the fact that they are the providers of salt to our table, the studies on their algal flora with potential presence of harmful species and relevant phycotoxin production is of significant importance. However, very few data on the topic are available.

Generally, cyanoprokaryotes are well developed in saltworks and salt lakes, occurring both in plankton and benthic mats at concentrations up to 200–250 g L^−1^ [7,319,380]. They are represented by both unicellular coccal algae like *Aphanothece halophila* and non-heterocytous filamentous *Coleofasciculus chthonoplastes* (Syn. *Microcoleus chtonoplastes*), *Halospirulina tapeticola* and *Phormidium* sp. [319,380]. Here we have to recall that *Aphanothece halophila* was found to produce nostocyclamide [142], which long was attributed solely to *Nosto*c [59].

Salterns exist also on the Bulgarian coast of the unique Black Sea, where the gradient starts from much lower point—16–18 g L^−1^ but reaches the same high concentrations of ca. 280 g L^−1^ [381]. During the last century, biodiversity of five different salt-work complexes showed similar pattern of cyanoprokaryote dominance with widely spread genera *Coleofasciculus* and *Lyngbya*, followed by *Spirulina* and *Phormidium* [382], all of which are known as toxigenic (Table 1). In some of these salterns the green macrophytic algae from *Ulva* and *Cladophora* reached high abundance [381], which is important due to growing evidence for allelopathic properties of *Ulva* [383].

##### Toxigenic Algae of Saline-Alkaline Environments

Saline alkaline environments (lakes and soils) are characterized by high total ion content (>60 g L^−1^ total salinity) and high pH > 9.5–11, widely known by soda systems due to simultaneous action of sodium carbonate and sodium bicarbonate [13]. Saline-alkaline soils are natural or man-made, formed by intensive irrigation/evaporation cycles, while most of soda lakes are land by origin (atahalassohaline), and their most diverse inhabitants are prokaryotes [13]. Cyanoprokaryotes of the genera *Arthrospira*, *Cyanospira*, *Synechococcus* and *Synechocystis* are essential in soda lakes [13,384]. In the soda lakes of Rift Valley, Eastern Africa, series of studies caused by mortality of Lesser Flamingo revealed presence of cyanotoxins (MCs, ATXs) in samples from lake water and benthic algal mats, in the isolated algal cultures and in stomachs, livers and fecal pellets of dead flamingoes as well, allowing to suppose that they were possibly related with their die-off episodes [385,386,387,388,389,390,391]. The suspected causative agents were *Limnospira fusiformis* (Syn. *Arthrospira fusiformis*) (the main food resource of the flamingoes) and *Anabaena* and *Anabaenopsis* from lakes waters, as well as Oscillatoriales and *Synechococcus* from the nearby situated hot springs, which drain directly in the lakes [386].

In the peculiar magnesium sulfate dominated Hot Lake, Washington, with pH range 7–8.5 and salinity gradient from 100 g L^−1^ salts on the water surface and 400 g L^−1^ salts on the bottom, the streptophyte macrophytic alga *Chara* was attached to the bottom, while in the water column below 1 m cyanoprokaryotes from the genera *Anacystis*, *Gomphosphaeria*, *Oscillatoria* and *Plectonema* developed [13,392]. Considering that *Anacystis* currently is recognized as genus separate from *Microcystis* [393], we have to note that all these genera are potentially toxigenic (Table 1).

The comprehensive studies of cyanoprokaryotes of the shallow alkali marshes (conductivity range 500–3000 µS cm^−1^) of Belize in Caribbean region revealed rich phycoflora in the phytoplankton and metaphyton and abundant development of benthic mats, dominated by cyanoprokaryotes [394,395,396,397,398]. The biodiversity of these marshes, as well as the algal flora of similar habitats in Florida Everglades and Islas dos Aves near Venezuelan coast, was locally characterized by endemic morpho- and ecospecies with unusual high species richness of cyanoprokaryotes from all main types (coccal, filamentous non-heterocytous and filamentous heterocytous) [397,399,400]. Although the newly described species have not been investigated for toxicity, some of them belong to toxin-producing genera (e.g., *Anabaena*, *Gloeotrichia*, *Nodularia*, *Tolypothrix*), and therefore, it could be expected that future studies will reveal presence of phycotoxins in these specific habitats.

An interesting report on cyanotoxins comes from investigation of water samples collected from several saline-alkaline lakes located at Centenário farm in the southern part of the sub-region Nhecolândia Pantanal wetland area in the north of the municipality of Aquidauana, Mato Grosso do Sul State, Brazil [193]. From a single benthic strain *Nostoc* sp. CENA543, isolated from a small, shallow lake in the region, a novel 6-membered family of linear tetrapeptides, pseudospumigins (Psp) was discovered [193]. Psp (Psp A-F) are new protease inhibitors close to other linear tetrapeptides, known from *Nodularia spumigena*, such as spumigins and Aers but are less potent, and the Psp gene cluster is more similar to the spumigin biosynthetic gene cluster than the Aer gene cluster [193]. The same *Nostoc* strain was found to be the first free-living NOD-producer from the genus *Nostoc.* Moreover, it produced NOD (desmethylNOD-R) at comparable levels as the toxic, bloom-forming, *Nodularia spumigena* with exceptionally high amounts of NOD-R. Therefore, studied wetlands were proved as a new environment in which the hepatotoxic NOD could be an adverse factor for human and domestic animal health. Of note, in ambient aquatic environments, besides the typical brackish waters, NODs were found also in freshwater lakes with *Nodularia spumigena* [32,401] and in a freshwater spring in tropical Australia (pH = 8.3, salinity 667 mg L^−1^), where the producer was the newly described cyanoprokaryote *Iningainema pulvinus* [402].

### 5.4. Toxigenic Radioresistant Algae

Ultraviolet (UV) and ionizing radiation (IR) are among the major threats to the Earth organisms. Few of them are adapted to live in higher than annual average of 2mGy og ionizing radiation, developing variety of preventing and repairing mechanism, including polyploidy [13]. The unicellular coccal cyanoprokaryote *Synechocystis* sp PCC68034 is able to accumulate large concentrations of Mn^2+^ [13]. The IR-resistance in the coccal cyanoprokaryote *Chroococcidiopsis* from desert and hypersaline environments possibly reflects its ability a to survive prolonged desiccation due to its thick protective mucilaginous (extracellular polysaccharide) envelopes and intracellular trehalose accumulation, as well as through efficient repair of the DNA damage that accumulates during dehydration [283,285,286]. There are no targeted toxin investigations on these radioresistant strains, but both genera have been reported as cyanotoxin producers [73,126] (Table 1).

### 5.5. Toxigenic Algae of Thermal Springs

Hot springs are well-isolated habitats which occur in clusters in globally distant regions and demonstrate clear patterns of phylogeny and distribution consistent with their geographical distribution [403,404]. Algae in these peculiar, “fascinating steady-state habitats” [4] (p. 203), are exposed to high constant temperatures but quite variable diurnal and seasonal insolation, with very limited grazing and insignificant seasonal differences and thrive mainly in structured benthic mats [4,405,406,407]. Cyanoprokaryotes, green algae and diatoms are the main contributors to the biodiversity in the vulnerable communities of the thermal springs [4,408,409]. No toxic green algae have been reported so far, but one of the classical inhabitants of thermal springs is the diatom *Amphora coffeaformis*, which currently is regarded as a DA-producer (Table 1) [410,411]. However, no data are available on the physical or chemical parameters triggering the synthesis of DA in this species [411].

Cyanoprokaryotes as primary producers comprise important part of the biodiversity and biomass of hot springs (e.g., [404,405,406,412,413,414]). Their polysaccharides and filaments were thought to present the backbone of the algal mats [407]. *Mastigocladus* is one of the most widespread genera in the thermal habitats, and therefore, it is worth to mention that it is phylogenetically close to the genus *Fischerella* [415], in which the cytotoxic antifungal fisherindole L [416,417], ambiguine isonitriles A–F, hapalindoles (G, H, L), fungicidal hapalindoletype alkaloids, ambigol A, B and indole alkaloid tjipanazole D, fischerellin A and MCs (-LR, -LA, -LF, -FR and demethyl-MC-LR) with their synthase genes (*mcyA-J*) were found [77,418,419]. In particular, in *Fischerella* strain PCC 7521, isolated from Yellowstone hot spring, free BMAA and protein BMAA were found [126]. Recent study suggested that hapalindoles should be added to the growing number of neurotoxins such as SXTs and ATXs [420]. Years ago, it was stressed that the presence of cyanoprokaryotic mats in hot springs may present health risk to humans through skin contact or due to accidental ingestion of contaminated water during recreational or bathing activities [421].

The first and best-known report on phycotoxins in thermal springs concerned NOD and its specific variant, a cyclic pentapeptide, [L-Homoarginine2] NOD, or [L-Har2], detected in the strain *Nodularia* PC7804 isolated from a thermal spring in France [422]. The parallel work on the same strain led to the simultaneous description of the same compound [423]. This important strain was firstly named as *N. harveyana*; later, it was revised as *N*. *sphaerocarpa*, but then, it was retained as strain PC7804 which is genetically distinct from both abovementioned species [424].

Reports on eye and skin irritations in people who used the Gazan hot springs of volcano origin in Saudi Arabia, where cyanoprokaryote mats developed, led to their investigation [425]. In a targeted study of two of these springs (Beni Malik, 48 °C and Al Bozah, 52 °C), 16 species were identified, but according to ELISA and HPLC analysis, only two of them, *Oscillatoria limosa* and *Synechococcus lividus*, were able to produce MCs (Table 1), and the values obtained exceeded the WHO limit for recreational waters [425]. In the same study, LPS were revealed for first time in hot springs by LAL assays with highest activity in the cultures of *Calothrix thermalis*, *Fischerella thermalis*, *Microcoleus vaginatus*, *Schizothrix calcicola* and *Synechococcus lividus* [425].

One of the best-known studies on harmful thermal algae was conducted in the hot springs on the shore of Lake Bogoria (Kenya) in relation with periodical mass mortality of Lesser Flamingos, which threatened their populations in the African Rift Valley [385,386,390,426]. This was the first report on the occurrence of cyanotoxins—MCs -LR, -RR, -LF and -YR and ATX in hot springs in thermal algal mats dominated by four cyanoprokaryotes, *Oscillatoria willei*, *Phormidium terebriformis*, *Spirulina subsalsa* and *Synechococcus bigranulatus*. Cyanotoxins in diverse concentrations were found in the tissue and stomach content of dead flamingos [385,386,390]. The presence of abovementioned toxins but in significantly (about 20-fold) lower concentrations in the same springs was detected four years later [386]. The same four species dominated in the hot springs of Manyara (Tanzania) where only MCs (0.1 µg L^−1^) were detected [386]. MCs in low amounts (0.2 µg L^−1^) were found in the samples the Hell’s Gate hot springs (Tanzania), where *Pseudanabaena galeata* dominated [386]. In Natron hot springs (Tanzania), in which *Cyanobacterium minervae* dominated in the shoreline mats, neither MCs nor ATX was detectable [386]. In Magadi hot springs (Kenya), where *Phormidium* sp. dominated, only MC (n.d. −0.5 µg L^−1^) in low concentrations were detected in the samples, but MC-LR in toxicologically relevant concentrations (0.4–0.8 µg g^−1^ FW) was found in the endemic fish *Oreochromis grahami*, which feed on cyanoprokaryotes [386].

Molecular-genetic screening for toxigenic genes in epilithic samples from three hot springs (Caldeira das Furnas, Furnas Hot Spring and Caldeira Velha) on S. Miquel Island (Azores, Portugal) revealed presence of genes for MC (*mcyA* and *mcyE*) and CYN production (pks and ps), but the presence of toxins was not investigated [427]. Only *mcyA* was detected in Caldeira das Furnas spring (pH = 8.7 and 54 °C), and all searched MC and CYN genes were found in Furnas Hot Spring (pH = 6.4 and 40 °C) [427] while no cyanotoxin synthase genes were registered in Caldeira Velha (pH = 4.6 and 30 °C); according to 16S rDNA sequences, six sequences similar to unidentified uncultured strains from hot vents in Mexico and Hawai were detected, and three more genera were reported: *Microcoleus*, *Pseudanabaena*, and *Gloeothece* [427]. The comparison of *mcy* and *pks* findings with 16S species identification showed presence of *Microcoleus* and *Pseudanabaena* in samples, from which MC and CYN synthase genes were found [427].

The total number of algae of thermal springs of Bulgaria, known for its geothermal activity and diversity of hot springs from different facies, contained 241 taxa (110 of which from Cyanoprokaryota), found in Bulgarian 35 thermal spring systems during a period of 120 years [414,428]. Considering the increasing role of thermal waters in the daily life of modern Bulgarian society, during a current investigation of one of the complexes, used for balneotherapy, a comparison of the species list with current data on cyanotoxin producers revealed that 40 species (or 74% of all algae found) could be outlined as potential producers (separately or in combination) of MCs, NODs, ATX, HTX and SXTs [428].

The use of mineral thermal spring hot waters for recreation and curation is a traditional activity since Thracian and Roman times. Due to their great prophylactic and therapeutic potential, nowadays, thermal springs gain increasing popularity as “sanitas per aquam” (SPA) resorts. The financial value of hot springs long has been evaluated by the ability to attract tourists or to generate thermal energy [406], but currently, the biotechnological interest in their inhabitants is increasing together with the increasing safety requirements [24]. The studies cited above showed that despite inimical for many organisms, the hot springs can be suitable environment for the growth of toxigenic species and that in some cases toxin levels can exceed the health limits, which inevitably demonstrated the need for screening and monitoring of these extreme habitats.

### 5.6. Toxigenic Algae of Cold Habitats

Traditionally, the cryosphere is defined as consisting of all parts of the globe in which water occurs in solid phase (ice or snow). In total, about 85% of our Planet (including oceans) has a permanent temperature below 5 °C, ice covers about 13% of the Earth surface and snow about 35% of the land surface [429]. Nowadays, it is widely accepted that cryoenvironments exist primarily in polar and high alpine regions and include versatile habitats like, snow, glaciers, ice caps and sea ice, and permafrost, but also cold lakes and ponds with permanent ice cover, as well as peculiar subzero saline springs, warmed by geothermal energy and deep ocean zones [7,13]. In contrast to the prior view that they are almost sterile, these places are actively inhabited by organisms named cryophiles (or psychrophiles) and contribute significantly to global organic carbon [4,13,430]. Many algae are distributed across a variety of cold environments and have to thrive and cope not only with low temperatures but also with the strong insolation perturbated with UV, osmotic stress, dryness from the wind, as well as with a poverty of nutrients and liquid water [13,24,104,431,432].

There are pronounced differences in the diversity and biomass of algae in the heterogenous cryoenvironments (i.e., on and in the ice (marine and freshwater), on snow surface and on, in other substrata like rocks and soils) [13].

More than 100 green algae thrive on snow surfaces and some of them in suitable locations can proliferate and cause snow pigmentation [13]. Commonly, green algae from the division Chlorophyta, and particularly *Chlamydomonas*/*Chloromonas*/*Microglena* or *Raphidonema*, dominate and cause bloody-red or green snow, but also green streptophytes like *Mesotaenium berggrenii* and *Ancylonema nordenskjoldii* can develop in visible grey or purple layers [13,423]. Rarer, yellow snow was reported as formed by mass development of the golden ochrophyte algae from the genus *Ochromonas* [13,423]. Up-to-now, the last one is the only recorded potential toxin-producing genus on the snow surface (Table 1).

In contrast to their sparse distribution on snow, ochrophytes (chromophytes) and particularly diatoms from genera *Aulacoseira*, *Fragillariopsis* and *Navicula* often dominate in freshwater and sea ice habitats, while cyanoprokaryotes (mainly filamentous) dominate in algal mats in high alpine or polar regions [13,315]. Peculiar living layers of cyanoprokaryotes, stromatolites, were found in the permanently ice-covered alkaline Antarctic Lake Untersee (pH 9.8–12.1) in the interior of the Gruber Mountains of central Queen Maud Land [433]. Domination of cyanoprokaryotes was proved also for the bottom of very specific psychrophilic habitats, distributed globally in glacier environments-cryoconites [13,434,435,436,437,438]. They are ice holes in glacial ablations with relatively low albedo due to accumulated dark particles, in which freezing and thaw phases are altering [13,437,438]. Although biological information on these transitional freshwater-ice habitats is still scarce, it has been supposed that generally cryoconites contained communities similar to that from lakes in the nearest valleys [104]. No specific research on toxins was done, but it has to be noted that most of the cyanoprokaryote genera identified in cryoenvironments have already been reported as toxigenic in freshwater habitats: *Geitlerinema*, *Leptolyngbya*, *Microcoleus*, *Nostoc* and *Phormidium* (Table 1).

In the harsh conditions of polar regions, much of the productivity comes from biocrusts dominated by filamentous cyanoprokaryotes from genera *Anabaena*, *Leptolyngbya*, *Lyngbya*, *Nostoc*, *Nodularia*, *Oscillatoria* and *Phormidium*, as it was revealed by studies in aquatic habitats (freshwater or brackish to saline meltwater ponds) of Antarctic [105,313,439,440,441]. These genera are well-known as cyanotoxin producers in different freshwaters (Table 1). Therefore, the finding of MCs (MC-LR) in benthic mats collected from Antarctic meltwater ponds [105,313,316,441,442] was expected. Other MCs (MC-FR, and MC-RR with some glycine modifications) were detected in ponds and lakes biofilms from distant geographic locations of Antarctic [105,440,441], and *Oscillatoria*, *Nostoc* and *Phormidium* were supposed as potential MC-producers [208,313]. Currently, MCs and BMAA were detected in preserved 100-year-old mats collected from Ross Island and McMurdo Ice Shelf during the legendary Captain R.F. Scott’s Discovery Expedition [443] and a new cyanoprokaryotic MC-producer, containing *mcyE* gene, *Anagnostidinema pseudacutissimum*, was discovered in a pond mat collected 35 years ago from Cape Crozier, Ross Island [444]. These findings confirm that MCs, BMAA and its isomers AEG and DAB are preserved under dry herbarium conditions [443] in accordance with the earlier reports on detectable MCs in herbarium specimens collected 60–170 years ago from temperate and tropical environments [445]. Considering all these data and also the results on aeroterrestrial algae from polar deserts, discussed in the beginning of the review, we agree with the opinion that the occurrence of cyanotoxins can exert a long-term impact on organisms co-existing in biocrust communities and can have far-reaching consequences for the entire polar ecosystem [315]. Moreover, the findings of cyanotoxins in almost all habitats of polar regions (with the fastest rates of recent climate warming) are of special importance since recent laboratory studies suggested that MCs are not only present in these ecosystems but also could increase with prolonged elevated water temperatures due to either preferential growth of toxigenic species or increased MC biosynthes [442].

## 6. Discussion and Conclusions

**Figure 1:**
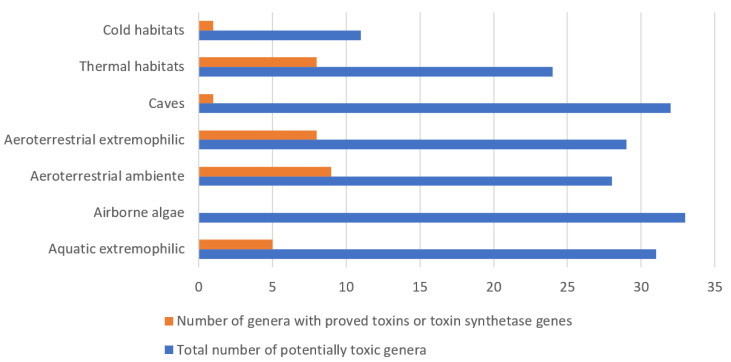
Number of potentially toxic genera in aeroterrestrial and extremophilic habitats compared with the number of genera from relevant habitats in which phycotoxins or toxin synthetase genes have been proved.

Although yet many aspects of knowledge on toxic aeroterrestrial, airborne and other extremophilic algae and their toxins remain obscure, an increasing number of surveys have addressed the topic. More evidence has been accumulated on the frequency of occurrence and concentrations at which phycotoxins are found, indicating the strong prevalence of the first oxygen-producing phototrophs on the Earth, cyanoprokaryotes, as toxigenic species and of cyanotoxins in all discussed ecological groups and versatile habitats (except for snow and ice). The exceptional taxonomic diversity of toxigenic species among airborne algae is caused by their origin in other ecological groups and cannot be related with the atmospheric environment. It has to be noted that similarly to the cases of ambient aquatic habitats, in the aeroterrestrial and other extreme habitats, just a low number of genera and species from the general vast biodiversity of the globe have been proved as toxigenic (Figure 1). According to the collected data in this review, only 47 genera of cyanoprokaryotes, five of dinoflagellates, and one from each of the groups of green algae, golden algae and diatoms can be related with phycotoxin and TOC production in different discussed habitats (Table 1). Among them, *Nostoc* stands out with the highest number of isolated and identified toxic compounds and seems to be widespread in almost all environments. Although some speculations can be made due to the great potential of this genus to occupy different habitats in both free-living and symbiotic stage, we shall not discuss it here, considering that the low number of studies on the other genera may be the simplest explanation for the case. A good example is the increasing number of toxic compounds found and clarified by mode of action in the genus *Fischerella*, spread in both ambient and extreme habitats, which started to attract the attention of different scientists since the early 90s of 20th century with increasing number of publications. A similar statement can be made, and the same explanation can be supposed, for the highest amount of toxins and toxigenic genera detected in all aeroterrestrial habitats (and in polar regions and deserts specifically). In contrast, evidence for toxin occurrence in caves is extremely rare and is practically lacking for snow and ice surfaces.

The analyzed ecological groups of aeroterrestrial and other extremophilic algae are specific per se and offer some specific compounds or their highly unusual types (nostosins, Nd A, Aer-865, Ap NZ857, MVs N3-N9, muscoride A, etc.) and even when “standard” phycotoxins have been also detected, often they occurred in specific structural variants with more congeners identified, which can be coupled with the harsh conditions, in which these organisms have to thrive (e.g., extremely low or high temperatures, strong thermal fluctuations, extreme aridity, high levels of solar and UV radiation). Interestingly, species from different ecological groups and with different mode of life were found to produce similar toxic compounds. A good example is nostocyclamide, found in aeroterrestrial/freshwater *Nostoc* and planktonic halotolerant *Aphanothece halophila*. Moreover, it was demonstrated that many of the studied strains produce more than one toxin type. However, their share and the supposed relations between diversity of toxins and environmental factors have to be clarified. On the other hand, analysis of collected data revealed the great potential of aeroterrestrial, airborne and other extremophilic algae to be an interesting source of novel compounds, and of toxic compounds with unique structures and wide spectrum of biological activities indicate their potential significant application in biotechnology, medicine and agriculture. Despite the meager data, the increasing number of findings of toxigenic algae and phycotoxins in environments different from the ambient aquatic habitats with increasing significance of inhalation and skin contacts as exposure routes and tracing of their accumulation in the food chains shows the clear need for studies geared at addressing this topic. In this way, the field is wide opened for many scientific disciplines. The fact that toxigenic algae and phycotoxins are widespread in a variety of habitats indicates how important is their screening and can serve as alarm for the need to intensify the studies, and the metabolomic studies in particular, at global scale in order to prevent many sanitary and environmental problems. Some studies showed the potential of genome-based discovery (“genome mining”) of natural products and the more intensive use of culture collections in such types of studies together with pure ecological and physiological ones has to be expected.

To conclude, despite the increasing interest to toxins of aeroterrestrial and other extremophilic algae, some aspects yet are either unexplored or underexplored, which allows us to outline the gaps in knowledge and to propose some ideas for future research. In the first place, there is a striking need of correct identification of toxin-producers based on polyphasic approach and conducting of simultaneous taxonomic and biochemical studies. This need is evident from the analysis provided above, in which most of the producers have not been accurately identified to species level. Another hinderance for analyses and future comparisons comes from insufficiently described sampling sites, and in this regard, we would like this review to serve as a plea for providing more detailed data on the localities or habitats. Second, even the scanty data on toxins and their producers in extreme habitats inevitably showed their presence in all general types of extremophile habitats. This shows the need for broad screening of phycotoxins in all these environments which will allow an overall view on their distribution and will help to outline the potential risks from recreational and curative activities, as well as from the consumed food and food additives. Parallel collection of data on coexisting or disparate toxic and non-toxic strains from the same species may help to elucidate their advantages or disadvantages in attempts to discover the biological role of phycotoxins. Third, comparison of the spread of different phycotoxins in such contrasting habitats like water and air may help to clarify the functional role of the toxic compounds and their evolutionary advantages. It is to recall that diverse taxa from the same taxonomic group can produce similar phycotoxins (Table 1), but also the same phycotoxins can occur in different evolutionary lineages, which is of phylogenetical interest. For example, similarity exists between toxins in eukaryotic red algae and diatoms (e.g., DA), but also some phycotoxins from prokaryotic and eukaryotic algae are the same and even if not completely identical, they have similar targets and modes of action (e.g., SXTs, CRs, Nd A and neurymenolide A). Fourth, considering the problems with time-consuming algal cultivation and difficulties of collection and transportation from the extreme habitats, we have to stress that every improvement in this field will be more than welcome. Fifth, the meager data on importance of symbiotic relations for production of phycotoxins for creation of resistance or tolerance against them allow to suppose this topic as significant in future studies. Sixth, the discovery of novel compounds, or at least novel variants of known biomolecules, has a great potential for medicine, pharmacy, cosmetics, bioremediation, agriculture and many other fields related with biotechnology. In this respect, the potential of aeroterrestrial and extremophilic algae is really untapped. It is not necessary to repeat in detail all aspects of the studies needed for revealing the driving forces of phycotoxin production and their importance in regard of increased eutrophication and global warming and associated health risks from the detrimental effects of harmful algae. However, we would like to stress that even for some important toxins and their producers, the driving forces are almost not clarified. We realize that this review more provokes questions than provides answers, and therefore, we believe that it will inspire an “extremophile” interest of more researchers in the algae from all non-ordinary habitats.

## Figures and Tables

**Table 1:** Toxin-producing genera and their toxic compounds (or toxin synthetase genes) in the groups of aeroterrestrial and other extremophilic algae. Legend: Aeroterr.—aeroterrestrial, Envm—environments, T/G—toxic compound or toxin synthetase gene (in cases when only genes have been reported), OC—occurrence in the relevant ecological group, ?—finding supposed. Aquatic ambient habitats are included since most toxins have been proved only in freshwater, brackish or marine environments. For toxin abbreviations and other explanations, see the text of the paper.

Algal Phylum/Genus	Toxin/Toxins and TOCs	Aquatic Ambiente Envm		Aquatic Extreme Envm		Airborne		Aeroterr. Ambiente Envm		Aeroterr. Extreme Envm		Thermal Envm		Cold Envm		Caves	
		T/G	OC	T/G	OC	T/G	OC	T/G	OC	T/G	OC	T/G	OC	T/G	OC	T/G	OC
Cyanopro- Karyota																	
*Anabaena* s.l.	MC, CYL; BMAA, spumigin J, Laxaphysins A-E; Geo	x	x		x		x	x	x		x		x				X (?)
*Anabaenopsis*	MC	x	x		x		x										
*Anagnostidinema*	MC													x	x		
*Anacystis*	MC (?)	x	x		x								x				X (?)
*Aphanocapsa*	MC-LR, NOD; MIB	x	x		x		x				x		x				x
*Aphanothece*	nostocyclamide, NOD	x	x	x	x		x		x		x		x				x
*Calothrix*	BMAA, LPS; Geo						x	x	x		x		x				x
*Chlorogloeopsis*	BMAA	x	x														x
*Chroococcidiopsis*	BMAA	x	x								x						x
*Chroococcus*	MC (?)		x	x (?)	x		x		x	x (?)	x		x		x		x
*Coleofasciculus*	MC, BMAA, DAB, AEG, GTX				x		x			x (?)	x						x
*Cyanobium*	MC (*mcyA* gene), CYN (*pks* gene)	x	x		x		x						x				x
*Cyanospira*	MC	x	x		x												
*Cylindrospermum*	Geo						x	x	x		x		x				x
*Fischerella*	MCs (MC-LR, MC-LA, MCLF, MC-FR and demethyl-MC-LR), BMAA, fisherindole L, ambiguine isonitriles A–F, hapalindoles (G, H, L), fungicidal hapalindoletype alkaloids, ambigol A, B and indole alkaloid tjipanazole D, fischerellin A, LPS; Geo	x	x				x	x	x		x	x	x				x
*Geitlerinema*	MCs, SXTs; mitsoamide	x	x								x		x		x		x
*Gloeocapsa*	MC	x	x				x				x		x		x		x
*Gloeotrichia*	MC-LF, MC-RR	x	x		x												
*Gomphosphaeria*	MCs	x	x		x		x										
*Hapalosiphon*	MCs (MC-LA), hapalosin	x	x		x		x	x	x		x						x
*Iningainema*	NOD			x	x												
*Komvophoron*	MCs, CYN (?)	x	x								x						x
*Leptolyngbya*	MCs; Geo	x	x				x				x		x		x		x
*Limnospira*	MC-YR; ATX	x	x		x		x										
*Lyngbya*	SXTs, CYN, deoxy-CYN, BMAA, LAs (A-C), AT, DAT	x	x		x		x				x		x		x		?
*Merismopedia*	MCs, NOD	x	x		x		x										
*Microchaete*	A90720A (protease inhibitor)						x	x	x				x				x
*Microcoleus*	MC (*mcyA*, *mcyE* genes), ANAs, BMAA (?), CYN (*pk*s genes), LPS; GEO				x		x	x (?)	x	x (?)	x	x	x		x		x (?)
*Microcystis*	MCs, ATXs, BMAA, MV-J, kawaguchipeptin-B	x	x		x		x						x (?)				x (?)
*Nodularia*	NOD, [L-Har2] NOD, BMAA, spumigins	x	x		x		x				x	x	x				
*Nostoc*	MCs, NOD, NOD-R (desmethylNOD-R), ATX, SXT, BMAA, Aer-865, Aps, banyasides, CPs, Cr, CV-N, MVs, Ncps, Nos, Ns, nostocyclamide, Nd A, nostophycin, nostoweipeptins (W1-W7), nostoginins, nostopeptins, insulapeptolides, nostocyclins, microginins, muscaride A, Psp A-F; Geo	x	x	x	x		x	x	x	x	x				x		x
*Oscillatoria*	MCs (-LR, -RR), ATX, HTX, CYN, 7-epi-CYN, DAT; Geo	x	x		x		x	x	x	x (?)	x	x	x		x		x
*Phormidium*	MCs, ATX, HTX, BMAA	x	x		x		x			x (?)	x	x			x		x
*Planktotohrix*	MCs, ATXs, BMAA, oscillatorin (oscyllamide Y), oscillapeptin	x	x														
*Plectonema*	MCs, BMAA	x	x		x						x		x		x		x
*Pseudanabaena*	MC-LR, MC (*mcyA* gene), CYN (*pks* gene)	x	x	x	x		x				x	x	x				x (?)
*Pseudocapsa*	MC-RR, MC-YR									x	x						
*Schizothrix*	DAT, LPS, schizothrin A	x	x				x	x	x		x	x	x		x		x
*Scytonema*	MC-LY, SXT, BMAA				x		x				x		x		x	x	x
*Snowella*	MCs	x	x				x										
*Spirulina*	MCs, ATXs				x		x					x	x				
*Symploca*	BMAA	x	x		x				x		x		x				x
*Synechocystis*	MC (incl. MC-LR), BMAA, LPS	x	x		x		x				x		x				
*Synechococcus*	MC, microcin-C like, NOD, BMAA, LPS, Thionsulfolipid	x	x		x		x				x	x	x				x
*Tolypothrix*	Tb				x		x				x						x
*Westiellopsis*	MC, westiellamide	x	x				x	x	x								x (?)
*Woronichinia*	MCs	x	x		x		x										
Pyrrhophyta																	
*Gymnodinium*	SXT, endotoxin; ichyotoxin	x	x				x										
*Karenia*	PbTx, gymnocin (GC)	x	x				x										
*Ostreopsis*	PLTXs (osteozin D, ovatoxin A, ostreotoxin-1 and 3, mascarenotoxin A and B)	x	x				x										
*Peridinium* s.l.	ichtyotoxins incl. alkaloid similar to 12-methoxyibogamine	x	x				x				x						
*Prorocentrum*	OA, DPX, prorocentrolides (PLC), borbotoxin	x	x				x										
Chlorophyta																	
*Ulva*	hemolysins	x	x		x												
Ochrophyta																	
*Amphora*	DA	x	x				x				x		x				x
*Ochromonas*	ichthyotoxic, hemolytic, and antispasmodic activities	x	x		x										x		

## Data Availability

Not applicable.
